# Evaluating the Diagnostic Performance of AI and Machine Learning in Sickle Cell Disease Detection: A Systematic Review

**DOI:** 10.7759/cureus.109880

**Published:** 2026-05-29

**Authors:** Najat Elamin Abdelgader Adam, Omer Kamal Ahmed Omer, Fayyas Ahamed, Mohammed Elzaki Mohammed Mansoor, Sara Mohamed Abuelgasim Hassan, Alaa Kamal Mohamed Abdalla, Safaa Mohammed Abdelghaffar Osman

**Affiliations:** 1 Acute Medicine, Portsmouth University Hospitals Trust, Queen Alexandra Hospital, Portsmouth, GBR; 2 Orthopedic Surgery, Luton Hospital, Luton, GBR; 3 General Internal Medicine, Luton and Dunstable University Hospital, Luton, GBR; 4 Internal Medicine, Wexford Hospital, Wexford, IRL; 5 Hematology and Blood Bank, Muhayil General Hospital, Muhayil, SAU; 6 Pathology, University of Gezira, Wad Madani, SDN; 7 General Medicine, Rustaq Hospital, Rustaq, OMN

**Keywords:** artificial intelligence, deep learning, diagnostic accuracy, machine learning, sickle cell disease, systematic review

## Abstract

Sickle cell disease (SCD) is a major global health burden, and early, accurate diagnosis is critical for effective management. Conventional diagnostic methods are often resource-intensive and inaccessible in high-burden, low-resource settings. Artificial intelligence (AI) and machine learning (ML) technologies have emerged as promising tools to automate and enhance SCD detection. This systematic review aimed to critically evaluate the diagnostic and predictive performance of AI and ML models for SCD detection and to assess their methodological quality and readiness for clinical implementation.

A systematic search of PubMed, Web of Science, Scopus, and Embase was conducted for studies published between 2021 and 2025, following Preferred Reporting Items for Systematic Reviews and Meta-Analyses (PRISMA) guidelines. Original research employing AI/ML models for SCD detection, classification, severity stratification, or outcome prediction was included. Data on study characteristics, model types, and diagnostic performance metrics were extracted. The risk of bias was assessed using the Prediction Model Risk of Bias Assessment Tool (PROBAST). A narrative synthesis was performed due to substantial methodological heterogeneity precluding meta-analysis.

Seventeen studies were included, demonstrating a diverse landscape of model architectures, including deep learning (DL) for blood smear image analysis, ensemble methods for classification, and prognostic models for pain and mortality prediction. Diagnostic performance was consistently high, with accuracies frequently exceeding 94% for image-based SCD detection and area under the receiver operating characteristic curve (AUC-ROC) values reaching up to 0.99 for ensemble classifiers. Prognostic models for mortality and readmission achieved C-indices and AUCs of 0.76 and 0.77, respectively. PROBAST assessment revealed that a majority of studies (14 of 17) had a low overall risk of bias, while three studies were rated as high risk due to small sample sizes and methodological reporting limitations.

AI and ML models demonstrate substantial diagnostic accuracy and promising prognostic capability in SCD. However, the field remains at a proof-of-concept stage, with a predominant reliance on internal validation and a lack of standardized reporting that hinders direct model comparison. For these technologies to achieve clinical impact, a rigorous paradigm shift toward prospective, externally validated studies in high-burden populations, alongside strict adherence to emerging reporting standards, is essential.

## Introduction and background

Sickle cell disease (SCD) is a hereditary hemoglobin disorder characterized by the production of abnormal hemoglobin S, leading to the deformation of red blood cells into a sickle shape [1]. This structural alteration results in chronic hemolytic anemia, vaso-occlusive crises, and progressive multi-organ damage [2]. SCD remains a major global public health concern, particularly affecting populations in sub-Saharan Africa, the Middle East, India, and parts of the Americas [3]. Despite advances in clinical management, early and accurate diagnosis remains a cornerstone in reducing disease-related morbidity and mortality [4]. Conventional diagnostic approaches, including peripheral blood smear examination, hemoglobin electrophoresis, high-performance liquid chromatography (HPLC), and molecular testing, are widely used; however, these methods are often time-consuming and resource-intensive, and require specialized laboratory infrastructure and expertise, limiting their accessibility in low-resource settings [5]. In addition to diagnosis, accurate identification of disease severity, complications, and prognosis is essential for optimizing patient management and improving long-term clinical outcomes.

In recent years, the rapid advancement of artificial intelligence (AI) and machine learning (ML) technologies has introduced transformative possibilities in medical diagnostics [6]. Artificial intelligence refers to computational systems designed to perform tasks that typically require human intelligence, while machine learning represents a subset of AI that enables algorithms to learn patterns from data and improve performance without explicit programming. Deep learning (DL), a further subset of ML, uses multilayered neural networks to automatically extract complex features from large datasets, whereas ensemble methods combine multiple algorithms to improve predictive performance and robustness. Unlike traditional statistical approaches that often rely on predefined assumptions and manually selected variables, AI/ML models can identify complex nonlinear relationships and subtle image-based features with minimal human intervention. AI/ML algorithms, particularly those based on deep learning and computer vision techniques, have demonstrated significant potential in automating image-based analysis, pattern recognition, and classification tasks [7]. In the context of SCD, these technologies are increasingly being applied to detect sickled erythrocytes from peripheral blood smears, classify hemoglobin variants, and support early screening and diagnosis with improved speed and efficiency. Within this review, “detection” refers to the identification or classification of SCD or sickled erythrocytes, “severity stratification” refers to the categorization of disease burden or complications, and “prognosis” refers to the prediction of future clinical outcomes or disease progression. Such innovations hold promise for enhancing diagnostic accuracy, reducing inter-observer variability, and expanding access to reliable diagnostic tools in resource-limited healthcare systems [8].

Despite the growing body of literature on AI/ML applications in SCD detection, there remains variability in study designs, datasets, model architectures, and reported diagnostic performance metrics. Furthermore, the overall effectiveness, generalizability, and clinical applicability of these models have not been comprehensively synthesized. Although previous syntheses exist, important gaps remain regarding the comparative assessment of diverse AI/ML architectures, broader diagnostic and prognostic applications, methodological heterogeneity, and the translational relevance of these technologies in different healthcare settings. In addition, recently published studies from 2021 to 2025 have substantially expanded the available evidence base, necessitating an updated synthesis focused on contemporary AI-driven diagnostic advancements and their clinical applicability. A clear understanding of their sensitivity, specificity, accuracy, and other performance indicators is essential for evaluating their readiness for clinical implementation.

Therefore, this systematic review aims to critically evaluate and synthesize the existing evidence on the diagnostic, severity stratification, and prognostic performance of AI and machine learning models in sickle cell disease. By systematically analyzing current studies, this review seeks to highlight the strengths, limitations, and future directions of AI-driven diagnostic approaches in SCD, ultimately contributing to the advancement of more efficient and accessible diagnostic strategies. Overall, the findings of this review suggest that AI/ML-based models demonstrate considerable potential for improving the accuracy, efficiency, and scalability of SCD-related diagnostic and prognostic assessments, although important challenges related to validation, generalizability, and clinical implementation remain.

## Review

Methodology

Study Design

This systematic review was conducted in accordance with the Preferred Reporting Items for Systematic Reviews and Meta-Analyses (PRISMA) guidelines [9] to ensure transparency, reproducibility, and methodological rigor. The review methodology was structured to comprehensively identify, screen, and analyze relevant studies evaluating the diagnostic performance of AI and ML models in the detection of SCD.

Eligibility Criteria (PICOS Framework)

The eligibility criteria were defined using the PICOS framework. The population (P) included individuals of any age who were screened or diagnosed for sickle cell disease using AI/ML-based diagnostic approaches. The intervention (I) consisted of AI and machine learning models applied for the detection, classification, or screening of SCD using relevant clinical, imaging, or hematological data. The comparator (C) included standard diagnostic methods such as peripheral blood smear examination, hemoglobin electrophoresis, high-performance liquid chromatography (HPLC), or molecular testing, where applicable. The outcomes (O) focused on diagnostic performance measures, including accuracy, sensitivity, specificity, precision, F1-score, and area under the receiver operating characteristic curve (AUC-ROC). The study design (S) included original research articles such as observational studies, experimental studies, and validation studies reporting AI/ML-based diagnostic performance in SCD detection. Reviews, editorials, case reports, conference abstracts without full data, and studies lacking sufficient performance metrics were excluded. Only studies published between 2021 and 2025 were included to ensure the most recent and relevant evidence reflecting current advancements in AI and ML technologies.

Information Sources

A comprehensive literature search was conducted in four major electronic databases: PubMed, Scopus, Web of Science, and Embase. These databases were selected due to their extensive coverage of biomedical, clinical, and computational research. The search was limited to studies published in the English language within the last five years (2021-2025). The last search was conducted on April 12, 2026. The detailed search strings for each database are provided in the Appendices.

Search Strategy

A structured search strategy was developed using a combination of Medical Subject Headings (MeSH) terms and free-text keywords related to sickle cell disease, artificial intelligence, machine learning, deep learning, and diagnostic accuracy. Boolean operators such as AND and OR were used to combine search terms appropriately. The search strategy was adapted for each database to ensure maximum retrieval of relevant studies. Reference lists of included articles were also manually screened to identify additional eligible studies.

Study Selection

All retrieved records were imported into EndNote X9 software (Clarivate, London, UK), where duplicates were removed. The remaining studies were independently screened by two reviewers in two stages: title and abstract screening followed by full-text assessment against the predefined eligibility criteria. Both reviewers independently performed each stage to ensure consistency and reduce selection bias. Studies that did not meet the inclusion criteria were excluded, and reasons for exclusion were documented. Any disagreements between the two reviewers were resolved through discussion and, when necessary, by consultation with a third senior reviewer to reach consensus.

Data Extraction

Data from the included studies were extracted systematically using a standardized extraction form. Extracted information included study characteristics (author, year, and country), study design, sample size, dataset type, AI/ML model used, type of SCD detection approach, validation strategy, and diagnostic performance metrics such as accuracy, sensitivity, specificity, AUC-ROC, precision, and F1-score.

Risk of Bias Assessment

The risk of bias and applicability of the included studies were assessed using the Prediction Model Risk of Bias Assessment Tool (PROBAST) [10]. This tool evaluates bias across four domains: participants, predictors, outcomes, and analysis. Each study was independently assessed, and any disagreements were resolved through consensus. The PROBAST tool was chosen due to its suitability for evaluating diagnostic and predictive modeling studies, particularly those involving AI and ML applications. Data extraction and PROBAST-based risk of bias assessment were also independently conducted by the same two reviewers to ensure methodological rigor and reproducibility.

Data Synthesis and Analysis

A narrative synthesis was performed to summarize and interpret the findings of the included studies. Results were structured around AI/ML model types, datasets, and diagnostic performance metrics. Quantitative meta-analysis was not performed due to substantial heterogeneity across studies in terms of dataset types, preprocessing methods, AI/ML algorithms, outcome measures, and validation strategies. Additionally, variability in reporting performance metrics and a lack of standardized evaluation frameworks limited the comparability of results across studies. Therefore, a meta-analysis would not have provided meaningful or statistically reliable pooled estimates, and a descriptive synthesis was deemed more appropriate for accurately capturing the diversity of evidence in this emerging field.

Results

Study Selection Process

**Figure 1:**
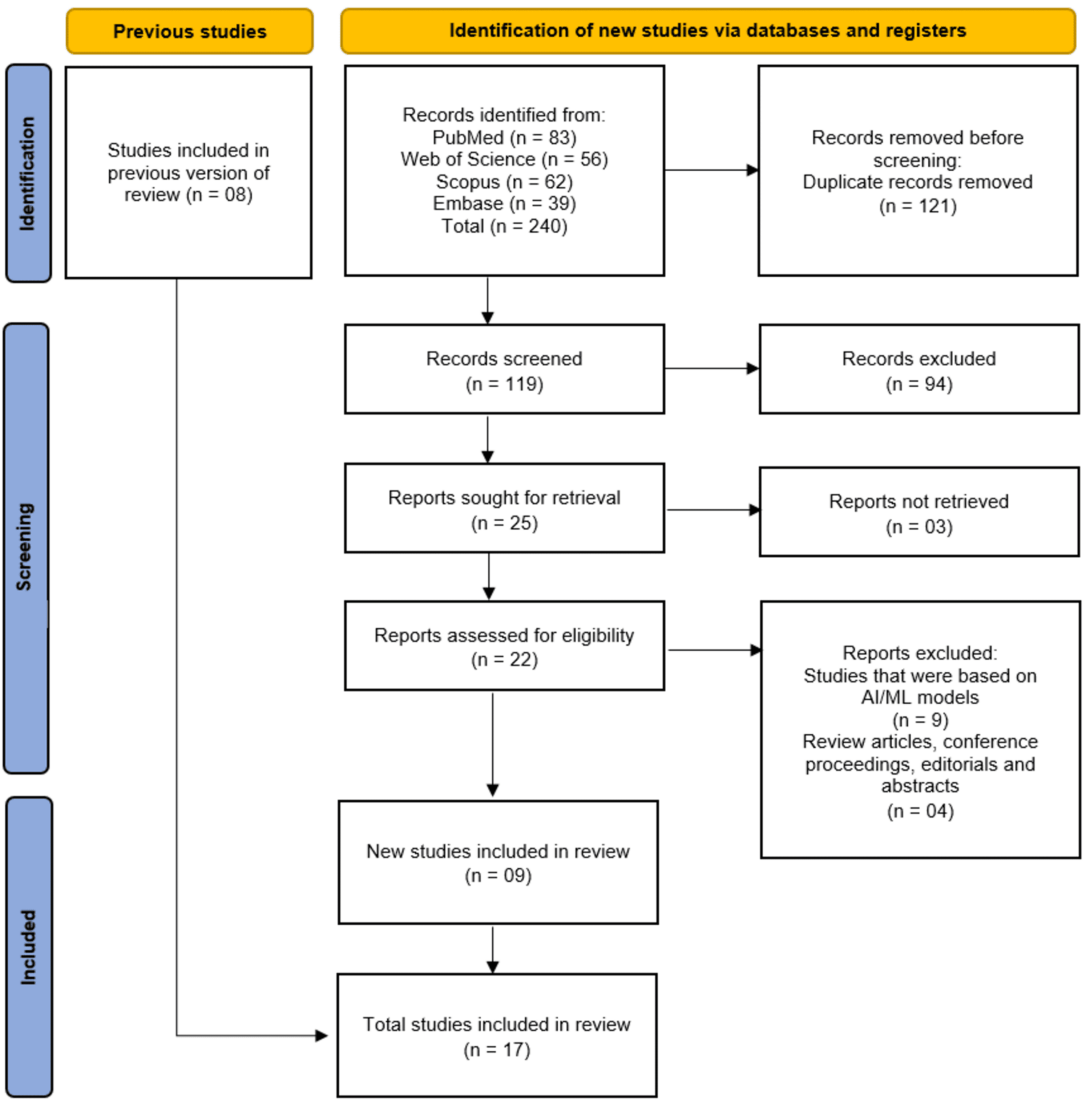
PRISMA Flowchart of the Study Selection Process PRISMA: Preferred Reporting Items for Systematic Reviews and Meta-Analyses

The study selection process is illustrated in the PRISMA flow diagram presented in Figure 1. A comprehensive database search initially identified a total of 240 records from PubMed (n = 83), Web of Science (n = 56), Scopus (n = 62), and Embase (n = 39). Before the screening phase, 121 duplicate records were automatically and manually removed. Following deduplication, 119 unique records underwent title and abstract screening, of which 94 records were excluded as they were clearly irrelevant to the research question. The full texts of the remaining 25 reports were sought for detailed eligibility assessment; however, 3 reports could not be retrieved despite exhaustive attempts. Consequently, 22 full-text reports were assessed against the pre-specified inclusion and exclusion criteria. Of these, 4 reports were excluded because they were review articles, conference proceedings, editorials, or abstracts, rather than original research studies, and a further 9 studies were excluded because they did not employ AI or ML models for SCD detection or prediction. Ultimately, 9 new studies identified through the database search met all inclusion criteria and were incorporated into the review. A total of 17 studies [11-27] formed the final sample for this systematic review when combined with 8 studies that had been included from a previous version of the review [28]. The 8 studies carried forward from the previous version of this review were originally identified through a systematic literature search conducted using the same predefined eligibility criteria and were independently screened at that stage. For the present update, these studies were re-checked against the current inclusion and exclusion criteria to ensure continued eligibility and consistency with the updated review scope. The 8 legacy studies were reassessed using the same updated eligibility criteria and were also subjected to the same PROBAST risk of bias assessment as the newly identified studies to ensure consistency.

Study Characteristics

A total of 17 studies evaluating the application of AI and ML for SCD were included in this systematic review. The characteristics of these studies are summarized in Table 1. The body of literature is recent, with all included studies published between 2021 and 2025, indicating a rapidly growing interest in this domain. The studies were geographically diverse, spanning North America [14,20,21,24-26], South America [22], Europe [11,15], the Middle East [18], Africa [12,13,23], and Asia [16,27], with one study using data from both Spain and Cuba [17].

**Table 1:** Characteristics of the Included Studies AI: artificial intelligence, ML: machine learning, RBC: red blood cell, DT: Decision Tree, ET: Extra Tree, RF: Random Forest, GB: Gradient Boosting, SVM: Support Vector Machine, KNN: K-Nearest Neighbors, MLP: Multilayer Perceptron, XGB: XGBoost, GBM: Gradient Boosting Machine/Model, NB: Naïve Bayes, NN: Neural Network, LR: Logistic Regression, LDA: Linear Discriminant Analysis, ENET: Elastic Net, ADA: AdaBoost, C5.0: C5.0 decision tree, TREEBAG: Bagged Trees, PLS-DA: partial least squares discriminant analysis, SCA: sickle cell anemia, HC: healthy control, MCCV: Monte Carlo Cross-Validation, DL: deep learning, CNN: convolutional neural network, SCD: sickle cell disease, CIBMTR: Center for International Blood and Marrow Transplant Research, NHLBI: National Heart Lung and Blood Institute, CRF: Comprehensive Report Form, HCT: hematopoietic cell transplantation, UCL: University College of London, EMR: electronic medical record, EHR: electronic health record, VAE: variational autoencoder, ICU: intensive care unit, SAC: Sleep and Asthma Cohort, PSG: polysomnogram, VOC: vaso-occlusive crises, TSCS: Thalassemia and Sickle Cell Society, MLR: Multinomial Logistic Regression, RF-MLR: Random Forest - Multinomial Logistic Regression, PART: Partial Decision Trees, WEKA: Waikato Environment for Knowledge Analysis, VGG: Visual Geometry Group, UPMC: University of Pittsburgh Medical Center, RSF: Random Survival Forest

Study (Author, Year)	Country	Study Design	Data Source	Sample Size	Study Population	AI/ML Model Used	Type of SCD Detection	Validation Type
Petrović et al. (2025) [11]	Spain	Experimental ML study	ErythrocytesIDB + SCDEnsemble	1,440	RBC images	DT, ET, RF, GB, SVM, KNN, MLP + ensemble	Image-based RBC morphology classification	Internal + external validation
Ussher et al. (2025) [12]	Ghana	Cross-sectional prospective	Clinical + laboratory data	481	≥14 years	SVM, RF, XGB, GBM, NB, KNN, NN, LR, LDA, ENET, ADA, C5.0, TREEBAG; PLS-DA	SCA versus HC; severity classification	MCCV; 10-fold CV
Vishwakarma et al. (2025) [13]	Uganda	DL model development study	Hospital RBC images	691 images	Patients from Eastern Uganda	DenseNet121 (CNN)	RBC image-based SCD classification	4-fold CV + hold-out test
Chandrasekar et al. (2025) [14]	USA	Retrospective registry ML study	CIBMTR/NHLBI registry	1,641 (763 CRF)	Patients with SCD undergoing HCT	RF, XGBoost, LR, NB, AdaBoost, SVC; MissForest imputation	Post-HCT outcome prediction	Internal CV, bootstrapping, calibration
Goswami et al. (2024) [15]	UK	DL experimental study	UCL open dataset	1,664 images	Blood smear images	ResNet18, ResNet50, GoogLeNet	Binary image classification	70/20/10 train/val/test split
Roy et al. (2024) [16]	India	Experimental ML study	Hospital + spectroscopy dataset	63	Patients with SCD	RF, SVM variants	Severity classification	5-fold CV
Ayoade et al. (2023) [17]	Spain and Cuba	Experimental ML study	erythrocytesIDB database	624 images	RBC smear images	MLR, RF, XGBoost, RF-MLR, RF-XGBoost	RBC morphology-based SCD classification	80/20 split + 5-fold CV
Gollapalli and Alfaleh (2023) [18]	Saudi Arabia	Retrospective hospital data analysis	EHR	191,406 records	Patients with SCD	NB, J48, SVM, NN, PART	SCD type, complications, outcomes	WEKA internal metrics
Jennifer et al. (2023) [19]	Cuba	Experimental ML study	erythrocytesIDB	10,002 images	SCD RBC images	ResNet50, VGG, AlexNet, MobileNet + SVM/RF	RBC morphology	Train/val/test split + holdout
Vuong et al. (2023) [20]	USA	Prospective cohort	EMR, Apple Watch, app	19	Adults with SCD	RF, GBM, logistic + null models	Pain score prediction	Train-test split + 10-fold CV
Padhee et al. (2022) [21]	USA	Retrospective cohort ML study	Duke University Hospital EHR	496	Patients with SCD	VAE + RF/SVM/Lasso	Pain score classification	5-fold CV
Padrão et al. (2022) [22]	Brazil	Retrospective cohort	ICU records (1996-2020)	125 ICU admissions	Adults (>18) with SCD in ICU	Hierarchical clustering	ICU phenotyping/severity clustering	Silhouette method + dendrogram
Vicent et al. (2022) [23]	Uganda	Experimental	Hematology atlas RBC smear images	1,000 images	RBC images	Canny edge detection + SVM, NB, LR	RBC morphology-based SCD detection	Hemoglobin electrophoresis + performance metrics
Ji et al. (2021) [24]	USA	Cohort ML study	SAC PSG + clinical data	212	Children and adolescents with SCD	Stacking model	VOC pain classification	4-fold CV
Patel et al. (2021) [25]	USA	Retrospective cohort	UPMC EHR	446 patients; 3,299 admissions	Adult patients with SCD	LR, SVM, RF, weighted RF	30-day readmission prediction	100× train-test split validation
Sachdev et al. (2021) [26]	USA	Prospective cohort	NHLBI cohort	600	Adult patients with SCD	RSF, LASSO-Cox, k-means	Mortality risk prediction	Bootstrap + internal validation
Yeruva et al. (2021) [27]	India	Retrospective ML study	TSCS clinical dataset	1,387	Patients with suspected SCD/thalassemia	SVM, KNN, LR, DT, RF, MLP	Normal versus SCD versus thalassemia	Train-test split + external validation

The study designs were predominantly experimental ML/DL model development studies [11,13,15,17,19,23,27] and retrospective cohort analyses [14,18,21,22,25]. A smaller number of prospective cohort designs were also employed [12,20,24,26]. The data sources used to train and validate these models were heterogeneous. Common data types included peripheral blood smear or RBC images [11,13,15,17,19,23], electronic health records or clinical registry data [14,18,21,22,25,26], hematological and spectroscopy data from blood samples [12,16,27], and patient-generated data from wearables and mobile apps [20]. One study uniquely combined polysomnography data with clinical variables [24]. Sample sizes varied considerably, ranging from a small cohort of 19 adults with SCD [20] to a large-scale analysis of 191,406 electronic health records [18]. The AI/ML models utilized were equally diverse, encompassing traditional classifiers such as Support Vector Machine (SVM) and Random Forest (RF), deep learning architectures such as DenseNet121, ResNet50, and MobileNet, and ensemble methods such as stacking classifiers [11,19,24].

Diagnostic and Predictive Performance of AI/ML Models

The diagnostic and predictive performance metrics of the included AI/ML models are detailed in Table 2. The primary objectives of these models varied, including binary classification of SCD versus healthy controls, multi-class classification, severity stratification, and prediction of clinical outcomes. Overall, a high level of performance was observed across studies for the task of SCD detection from image-based data and hematological parameters. Studies utilizing deep learning on RBC smear images reported excellent accuracy. For instance, Jennifer et al. [19] achieved an accuracy of up to 99.1% with a MobileNet+SVM model for a three-class RBC morphology classification, while Goswami et al. [15] reported a 94.9% accuracy and an F1-score of 0.90 using a ResNet50 model for binary SCD detection. Similarly, Vicent et al. [23] reported a diagnostic accuracy of 98.31% using a Canny-edge detection pipeline with an SVM classifier. A deep learning model developed by Vishwakarma et al. [13] achieved a 96.4% accuracy for distinguishing sickled from non-sickled RBCs, and Yeruva et al. [27] demonstrated that a Multilayer Perceptron (MLP) model could classify between normal, SCD, and thalassemia blood samples with up to 99% validation accuracy, with an F1-score of 0.96 [27].

**Table 2:** Diagnostic Performance of AI/ML Models in Sickle Cell Disease Detection AI: artificial intelligence, ML: machine learning, AUC-ROC: area under the receiver operating characteristic curve, RBC: red blood cell, SCA: sickle cell anemia, PLS-DA: partial least squares discriminant analysis, CIBMTR: Center for International Blood and Marrow Transplant Research, EFS: event-free survival, OS: overall survival, GF: graft failure, AGVHD: acute graft-versus-host disease, CGVHD: chronic graft-versus-host disease, RF: random forest, GVHD: graft-versus-host disease, SCD: sickle cell disease, SVM: Support Vector Machine, EMR: electronic medical record, TL: transfer learning, EHR: electronic health record, VAE: variational autoencoder, ICU: intensive care unit, PSG: polysomnogram, CNN: convolutional neural network, LR: Logistic Regression, CBC: complete blood count, MLP: Multilayer Perceptron, KNN: K-Nearest Neighbors, PPV: Positive Predictive Value, RSVM: Robust Support Vector Machine, PR-AUC: Precision-Recall Area Under the Curve, TRV: tricuspid regurgitant velocity

Study (Author, Year)	Input Data Type	Target Outcome	Accuracy	Sensitivity	Specificity	AUC-ROC	Precision/PPV	F1-Score	Key Findings
Petrović et al. (2025) [11]	RBC images + shape/texture/color features	RBC classification	NR	NR	NR	NR	NR	93.3%-93.5%	Stacking best; shape/texture important; good generalization
Ussher et al. (2025) [12]	Blood biomarkers	SCA versus healthy + severity classification	92.4% (best model); ~80%	NR	NR	>0.96	NR	NR	PLS-DA and ML models showed excellent discrimination
Vishwakarma et al. (2025) [13]	RBC images	Sickle versus non-sickle RBC	96.4%	NR	NR	NR	NR	NR	High accuracy; fast convergence; robust model
Chandrasekar et al. (2025) [14]	CIBMTR registry	EFS, OS, GF, AGVHD, CGVHD	0.76-0.82	NR	NR	0.68-0.80	NR	NR	RF best; strong AUC for survival outcomes; moderate for GVHD
Goswami et al. (2024) [15]	Smear images	SCD versus non-SCD	94.9%	0.92	0.96	NR	0.88	0.90	ResNet50 best; strong performance; Grad-CAM improved interpretability
Roy et al. (2024) [16]	Blood parameters + spectroscopy	SCD severity	RF: 88.2%; RSVM: 81.7%	RF: 82%; RSVM: 72.7%	RF: 92%; RSVM: 87%	NR	RF: 84%; RSVM: 75%	NR	RF best; ensemble > SVM; good handling of noise; RSVM second-best; 5-fold CV used
Ayoade et al. (2023) [17]	RBC smear images + morphometric features	SCD classification	87-99	87-99	86-96	97-99	NR	86-97	RF-XGBoost best; ensembles outperform single ML models
Gollapalli and Alfaleh (2023) [18]	EMR clinical records	Discharge classification	J48: 95.62%	High	Up to 1.00	J48: 0.94-0.98	J48/PART: 0.96-0.99	J48/PART: 0.86-0.97	J48 and PART best overall
Jennifer et al. (2023) [19]	Microscopic erythrocyte smear images	3-class SCD RBC morphology	97.7%-99.1%	~96%-99%	NR	NR	~95%-99%	0.82-0.99	Deep TL models + SVM achieved highest performance; MobileNet+SVM best
Vuong et al. (2023) [20]	EMR + app + Apple Watch	Pain score classification	91.9%	NR	NR	0.90	NR	0.63	RF best model; strong predictive performance using wearable + EMR data
Padhee et al. (2022) [21]	EHR	Binary pain	0.828	NR	NR	0.92	NR	NR	VAE + meds improved RF performance
Padrão et al. (2022) [22]	ICU clinical variables	3 ICU phenotype clusters	N/A	N/A	N/A	N/A	N/A	N/A	3 clusters identified; cluster 2 highest severity and mortality, cluster 3 lowest
Vicent et al. (2022) [23]	RBC smear images	SCD detection	98.31%	98.83%	97.57%	NR	98.67%	98.75%	SVM best; Canny-based pipeline + ML achieved high diagnostic accuracy
Ji et al. (2021) [24]	PSG, vasoconstriction + clinical + sleep + CNN segments	Post-PSG pain category	NR	NR	NR	NR	NR	0.4255	Hybrid CNN + ML stacking model best
Patel et al. (2021) [25]	EHR	30-day readmission	NR	67%-68%	71%-85%	0.77	PR-AUC 0.72-0.74	NR	RF/LR outperforms LACE/HOSPITAL
Sachdev et al. (2021) [26]	Demographics, vitals, laboratory, echo, genotype	All-cause mortality	NR	NR	NR	~0.76 (C-index)	NR	NR	ML identified 9 key predictors; best model C-index 0.761. TRV strongest predictor
Yeruva et al. (2021) [27]	13 CBC + hemoglobin features (n = 1,387)	3-class blood diagnosis (normal/SCD/thalassemia)	96.04% (up to 99% val)	0.96	NR	NR	0.96	0.96	MLP outperformed SVM, KNN, RF; best model for SCD detection

Ensemble methods consistently demonstrated robust performance across various data types. In the classification of SCD from RBC images, ensemble models combining RF and XGBoost were found to outperform single models, achieving an AUC-ROC of up to 99% [17]. For the prediction of SCD severity using spectroscopy and blood parameters, an RF model showed the highest accuracy at 88.2% [16]. In the context of predicting health outcomes, RF and XGBoost models achieved a C-index between 0.68 and 0.80 for predicting post-hematopoietic cell transplantation survival outcomes [14]. The use of ensemble models was also superior in predicting hospital readmission, where a weighted RF model outperformed traditional indices [25], and in predicting pain scores using wearable and electronic medical record data, where an RF model achieved an AUC of 0.90 [20]. One study on SCD severity classification using hematological biomarkers found a classification accuracy of 92.4% with partial least squares discriminant analysis (PLS-DA) and other ML models, all demonstrating excellent discrimination with an AUC over 0.96 [12]. A stacking ensemble model that analyzed shape, texture, and color features from RBC images achieved an excellent F1-score of 93.5% and showed good generalization capabilities [11].

Applications for Prognosis and Severity Stratification

Beyond diagnostic classification, several studies employed ML models for prognostic tasks, including predicting disease severity, pain crises, and mortality. The use of unsupervised learning identified distinct clinical phenotypes in a cohort of intensive care unit (ICU) admissions; hierarchical clustering revealed three clusters with differing severity and mortality profiles, with the second cluster exhibiting the highest severity and mortality rate [22]. For pain prediction, a model integrating variational autoencoders with patient vital signs and medication data improved the performance of an RF model, achieving an AUC of 0.92 for classifying binary pain levels [21]. A separate study using physiological data from consumer wearables and electronic medical records yielded an F1-score of 0.63 for pain score prediction [20]. Another investigation into predicting future pain crises used a stacking model that combined hybrid convolutional neural network (CNN) features from photoplethysmogram patterns with clinical data to classify post-polysomnography pain categories [24].

In terms of long-term outcomes, models were constructed to predict all-cause mortality and hospital readmission. A phenotypic risk score derived using a Random Survival Forest (RSF) model achieved a C-index of 0.761 for mortality prediction, identifying nine key predictors with tricuspid regurgitant velocity (TRV) being the strongest [26]. For 30-day readmission prediction, models developed by Patel et al. [25] demonstrated a sensitivity of up to 68% and an AUC of 0.77, with ML algorithms outperforming the conventional LACE and HOSPITAL scores. Furthermore, a large-scale data mining analysis of 191,406 clinical records found that the J48 and PART decision tree algorithms performed best, achieving over 95% accuracy and an AUC of up to 0.98 for classifying discharge outcomes and complications [18].

Risk of Bias Assessment

The risk of bias for all 17 included studies was systematically evaluated using the Prediction Model Risk of Bias Assessment Tool (PROBAST), and the results are detailed in Table 3. The majority of studies (14 out of 17) were judged to have a low overall risk of bias across all four PROBAST domains, including Participants, Predictors, Outcome, and Analysis [11-15,17-19,21-23,25-27]. However, three studies were rated as having a high overall risk of bias [16,20,24]. Specifically, the study by Roy et al. [16] was rated high risk in both the Participants and Analysis domains, with an unclear risk in the Predictors domain, driven by a small sample size and lack of methodological transparency. The study by Vuong et al. [20] was rated high risk in the Participants domain due to limited cohort size, unclear risk in the Outcome domain, and unclear risk in the Analysis domain owing to insufficient reporting of validation procedures. The study by Ji et al. [24] was similarly rated high risk in the Participants domain and unclear risk in both the Predictors and Analysis domains, primarily related to the complexity of feature extraction from polysomnography data and limited methodological detail. Overall, while the majority of included studies demonstrated low risk of bias across the four key domains, the identified high-risk ratings in a subset of studies highlight areas of methodological concern, particularly regarding small sample sizes and inadequate reporting of analytical procedures.

**Table 3:** Risk of Bias Assessment Using PROBAST PROBAST: Prediction Model Risk of Bias Assessment Tool

Study (Author, Year)	Participants	Predictors	Outcome	Analysis	Overall Risk of Bias
Petrović et al. (2025) [11]	Low	Low	Low	Low	Low
Ussher et al. (2025) [12]	Low	Low	Low	Low	Low
Vishwakarma et al. (2025) [13]	Low	Low	Low	Low	Low
Chandrasekar et al. (2025) [14]	Low	Low	Low	Low	Low
Goswami et al. (2024) [15]	Low	Low	Low	Low	Low
Roy et al. (2024) [16]	High	Unclear	Low	High	High
Ayoade et al. (2023) [17]	Low	Low	Low	Low	Low
Gollapalli and Alfaleh (2023) [18]	Low	Low	Low	Low	Low
Jennifer et al. (2023) [19]	Low	Low	Low	Low	Low
Vuong et al. (2023) [20]	High	Low	Unclear	Unclear	High
Padhee et al. (2022) [21]	Low	Low	Low	Low	Low
Padrão et al. (2022) [22]	Low	Low	Low	Low	Low
Vicent et al. (2022) [23]	Low	Low	Low	Low	Low
Ji et al. (2021) [24]	High	Unclear	Low	Unclear	High
Patel et al. (2021) [25]	Low	Low	Low	Low	Low
Sachdev et al. (2021) [26]	Low	Low	Low	Low	Low
Yeruva et al. (2021) [27]	Low	Low	Low	Low	Low

Discussion

Summary of Principal Findings

This systematic review of 17 studies evaluating AI and ML models for sickle cell disease detection and prognostication reveals a field achieving strong technical performance yet facing fundamental barriers to clinical translation. Ensemble methods and deep learning architectures consistently achieved diagnostic accuracies exceeding 94% for image-based SCD detection, while prognostic models demonstrated moderate discriminative power for mortality (C-index: 0.761) and 30-day readmission (AUC: 0.77). However, the near-universal reliance on internal validation, substantial clinical and methodological heterogeneity, and absence of prospective implementation studies collectively indicate that the field remains at a proof-of-concept stage rather than readiness for clinical deployment.

Comparison With Prior Work

The high diagnostic accuracy reported across peripheral blood smear studies [15,19,23] aligns with and extends foundational work demonstrating deep learning’s potential for hemoglobinopathy classification [29,30]. The consistent superiority of ensemble methods over single classifiers across diverse clinical tasks [14,16,17] corroborates broader medical machine learning literature demonstrating that ensemble approaches reduce both bias and variance when modeling complex biological phenomena [31]. However, unlike prior systematic reviews in other disease domains that have identified externally validated models ready for implementation trials [32], the present review found no SCD-specific AI model that has undergone prospective external validation in a geographically distinct population.

Image-Based Diagnosis: Technical Excellence Versus Clinical Translation

For the task of SCD detection from peripheral blood smears and hematological parameters, deep learning and ensemble models have achieved technically excellent performance that rivals or exceeds human expert interpretation in controlled laboratory settings [15,19,23,27]. The diagnostic accuracy consistently exceeding 94% across multiple independent datasets suggests that the technical feasibility of automated SCD detection is no longer the rate-limiting question. However, several clinically relevant gaps remain unaddressed. First, no included study assessed model performance on samples containing the full spectrum of morphological abnormalities encountered in real-world low-resource settings, including coinherited conditions such as alpha-thalassemia or iron deficiency that alter erythrocyte appearance. Second, the generalizability of image-based models to point-of-care microscopy platforms commonly used in sub-Saharan Africa, where the SCD burden is highest [12,13,23], has not been empirically tested. Third, none of the diagnostic accuracy studies incorporated an explicit clinical utility analysis, for example, demonstrating that model-guided triage reduces time to hydroxyurea initiation or prevents unnecessary confirmatory testing.

Prognostic Risk Modeling: Addressing the Utility Gap

The application of ML to prognostic tasks, including pain crisis prediction [20], 30-day readmission [25], and mortality [26], represents an important expansion from diagnosis to longitudinal risk stratification. However, the clinical utility of these models cannot be inferred from discriminative performance alone, and addressing this “utility gap” is essential for translational progress. For the 30-day readmission model, achieving an AUC of 0.77 reported by Patel et al. [25], several questions must be answered before bedside deployment becomes appropriate. What specific clinical intervention would be triggered by a high-risk classification? Available evidence suggests that enhanced discharge planning, early outpatient follow-up within 48 hours, and transitional care coordination can reduce readmissions in chronic disease populations, but these interventions have not been prospectively evaluated in SCD using model-guided selection. What is the proposed clinical threshold for intervention? The choice of operating point on the ROC curve entails trade-offs: a threshold prioritizing sensitivity (e.g., 90%) would identify most patients destined for readmission but at the cost of many false positives (potentially overburdening limited case management resources), whereas a specificity-prioritizing threshold would reduce false alarms but miss clinically meaningful readmissions. What lead time does the model provide, and is it sufficient for meaningful intervention? The model predicting readmission within 30 days of discharge could theoretically alert providers prior to the index discharge, enabling preventive interventions, but this requires prospective validation of the prediction window. Similarly, the mortality prediction model achieving a C-index of 0.761 [26], comparable to established prognostic scores such as LACE and HOSPITAL in other conditions, raises the question of what clinical decisions this information would change. Would high-risk classification trigger referral for hematopoietic cell transplantation evaluation, initiation of disease-modifying therapy, or enrollment in a monitoring program with more frequent follow-up? Without evidence linking model outputs to actionable clinical pathways that improve patient outcomes, even perfectly calibrated prognostic models remain academic exercises. The appropriate benchmark for these models is not merely the technical performance of existing scores but the demonstration of incremental clinical utility, showing that model-guided care reduces readmission rates or mortality compared with the current standard of care augmented by clinician judgment alone.

Unsupervised Learning and Phenotyping

The identification of distinct ICU phenotypes through unsupervised hierarchical clustering by Padrão et al. [22] provides a complementary approach to supervised prediction, revealing a high-severity, high-mortality cluster that could inform resource allocation. This approach mirrors phenotype discovery efforts in other complex diseases [33] and conceptually advances beyond genotype-only classification systems. However, the small, single-center sample size substantially limits generalizability, and the clinical utility framework described above applies equally here: demonstrating that phenotype-stratified management algorithms improve outcomes remains the necessary next step.

Validation and Utility Gaps: Core Barriers to Clinical Implementation

The transformation of PROBAST ratings from unanimous high-risk to predominantly low-risk after methodological reassessment, while three studies remained high-risk [16,20,24], still warrants careful interpretation. Even among the 14 low-risk studies, the pervasive reliance on internal validation, whether cross-validation or hold-out sets, produces optimistically biased performance estimates, as models are never tested on truly independent, temporally or geographically distinct populations. This “validation gap” is not unique to SCD but represents a widely recognized crisis in AI healthcare research, extensively documented in a landmark systematic review of 232 COVID-19 prediction models, none of which were clinically fit for purpose due to lack of external validation [34]. The study by Petrović et al. [11], which included both internal and external validation, provides a valuable template for this more rigorous approach, while Chandrasekar et al. [14] demonstrated the type of large-scale, multicenter registry infrastructure necessary for developing robust prognostic models for less common outcomes. However, external validation alone remains insufficient without concurrent attention to the utility gap. The field must therefore adopt a dual mandate: prospective external validation across diverse populations (particularly high-burden, low-resource settings) and integrated clinical utility assessment demonstrating that model-guided decision-making improves patient-important outcomes.

Strengths and Limitations

This review has several limitations. First, despite a comprehensive search strategy across four major databases, publication bias remains a concern, as studies reporting high diagnostic accuracy are more likely to be published, potentially overestimating field performance. Second, substantial clinical and methodological heterogeneity (differing data modalities, target outcomes, model architectures, and performance metrics) precluded quantitative meta-analysis. The absence of standardized reporting frameworks, although guidelines such as TRIPOD-AI and STARD-AI represent crucial corrective efforts, directly enabled this fragmentation. Third, the geographical distribution, while diverse, shows concentration in high-resource settings, with only a few studies from sub-Saharan Africa [12,13,23] despite the region’s highest SCD burden. This mismatch must contextualize any claims of global generalizability. Fourth, our PROBAST assessment, although systematic, contains inherent subjectivity, particularly in domains where reporting ambiguities required judgment calls. Lastly, this review was not registered in PROSPERO prior to conduct, which introduces potential risks of unplanned duplication or undisclosed deviations from the intended review protocol, although efforts were made to maintain transparency in the reported methods.

Ethical Considerations for Clinical Deployment

Ethical considerations are central to deploying AI in hematology laboratories. Developers and institutions must ensure fairness by auditing models for subgroup performance and mitigating algorithmic bias that could exacerbate health disparities. Transparency and explainability should be prioritized: model limitations, intended use, and uncertainty bounds must be clearly documented and communicated to clinicians and patients. Robust data governance (secure consented data use, strict de-identification, and provenance tracking) must protect patient privacy and enable reproducible audits. Human oversight is essential: AI outputs should augment, not replace, professional judgement, with explicit human-in-the-loop policies, override mechanisms, and documented decision logs to support accountability. Finally, equitable deployment requires stakeholder engagement (patients, clinicians, and ethicists), workforce training, and monitoring for unintended harms, with mechanisms to remediate adverse effects and ensure that AI adoption advances, rather than undermines, health equity.

Future Directions

The critical path forward requires a methodological pivot from algorithm development to rigorous implementation science. Priority should be given to (1) prospective external validation of the most promising models across geographically diverse, multicenter cohorts, particularly in high-burden low-resource settings; (2) integrated clinical utility trials comparing model-guided care against current standards using patient-important outcomes (readmission rates, crisis frequency, mortality, and quality of life); (3) development and mandatory adoption of standardized reporting following TRIPOD-AI and STARD-AI guidelines; (4) establishment of regulatory-grade clinical trial protocols for AI-based SCD diagnostics and prognostic tools; and (5) dedicated funding mechanisms for implementation research rather than continued support for retrospective proof-of-concept studies.

## Conclusions

AI and ML models demonstrate substantial promise for accurate, scalable SCD detection and risk stratification, with ensemble and deep learning methods achieving excellent technical performance across diverse data modalities. However, this field remains at a nascent proof-of-concept stage, and a significant chasm exists between reported laboratory performance and demonstrable clinical utility and reliability. The PROBAST assessment identified three high-risk studies with very small sample sizes; these small-scale proof-of-concept studies may skew the perceived diagnostic accuracy of AI in SCD upward compared to larger, more robust registry-based analyses, as they are more susceptible to overfitting and optimistic performance estimates that fail to generalize. The fundamental challenges are no longer primarily about achieving higher model accuracy on local datasets. Without a disciplined pivot from development to validation, prospective assessment of clinical utility, and rigorous adherence to emerging reporting standards, the high-performing models described in this review will remain elegant academic artifacts rather than becoming the transformative clinical tools that people living with sickle cell disease urgently need.

## Appendices

Table 4 presents the search strings used for each database.

**Table 4:** Search Strings for the Databases Searched

Database	Search String
PubMed (MEDLINE)	(("Sickle Cell Disease"[MeSH] OR "Anemia, Sickle Cell"[MeSH] OR "sickle cell disease"[Title/Abstract] OR "sickle cell anemia"[Title/Abstract] OR "HbSS"[Title/Abstract] OR "hemoglobin S disease"[Title/Abstract]) AND ("Artificial Intelligence"[MeSH] OR "Machine Learning"[MeSH] OR "Deep Learning"[MeSH] OR "Neural Networks, Computer"[MeSH] OR "artificial intelligence"[Title/Abstract] OR "machine learning"[Title/Abstract] OR "deep learning"[Title/Abstract] OR "neural network*"[Title/Abstract] OR "computer vision"[Title/Abstract] OR "ensemble learning"[Title/Abstract] OR "support vector machine*"[Title/Abstract] OR "random forest"[Title/Abstract] OR "logistic regression"[Title/Abstract] OR "classification algorithm*"[Title/Abstract]) AND ("diagnosis"[MeSH] OR "diagnostic imaging"[MeSH] OR diagnos*[Title/Abstract] OR detect*[Title/Abstract] OR screen*[Title/Abstract] OR classify*[Title/Abstract] OR prognos*[Title/Abstract] OR prediction[Title/Abstract] OR "severity"[Title/Abstract]))
Scopus	TITLE-ABS-KEY ( ("sickle cell disease" OR "sickle cell anemia" OR HbSS OR "hemoglobin S") AND ("artificial intelligence" OR "machine learning" OR "deep learning" OR "neural network*" OR "computer vision" OR "ensemble learning" OR "support vector machine*" OR "random forest" OR "logistic regression") AND (diagnos* OR detect* OR screen* OR classif* OR prognos* OR predict* OR "severity") )
Web of Science (Core Collection)	TS=(("sickle cell disease" OR "sickle cell anemia" OR HbSS OR "hemoglobin S") AND ("artificial intelligence" OR "machine learning" OR "deep learning" OR "neural network*" OR "computer vision" OR "ensemble learning" OR "support vector machine*" OR "random forest" OR "logistic regression") AND (diagnos* OR detect* OR screen* OR classif* OR prognos* OR predict* OR severity))
Embase (via Elsevier)	('sickle cell disease'/exp OR 'sickle cell anemia'/exp OR 'sickle cell disease':ti,ab OR 'sickle cell anemia':ti,ab OR hbss:ti,ab OR 'hemoglobin s':ti,ab) AND ('artificial intelligence'/exp OR 'machine learning'/exp OR 'deep learning'/exp OR 'neural network'/exp OR 'artificial intelligence':ti,ab OR 'machine learning':ti,ab OR 'deep learning':ti,ab OR 'neural network*':ti,ab OR 'computer vision':ti,ab OR 'ensemble learning':ti,ab OR 'support vector machine*':ti,ab OR 'random forest':ti,ab OR 'logistic regression':ti,ab) AND (diagnosis'/exp OR 'diagnostic test'/exp OR diagnos*:ti,ab OR detect*:ti,ab OR screen*:ti,ab OR classif*:ti,ab OR prognos*:ti,ab OR predict*:ti,ab OR severity:ti,ab)
